# Real-Time Embedded Eye Image Defocus Estimation for Iris Biometrics

**DOI:** 10.3390/s23177491

**Published:** 2023-08-29

**Authors:** Camilo A. Ruiz-Beltrán, Adrián Romero-Garcés, Martín González-García, Rebeca Marfil, Antonio Bandera

**Affiliations:** Departamento Tecnologia Electronica, ETSI Telecomunicacion, University of Málaga, 29071 Málaga, Spain; camilo@uma.es (C.A.R.-B.); argarces@uma.es (A.R.-G.); martin@uma.es (M.G.-G.); rebeca@uma.es (R.M.)

**Keywords:** eye detection, Haar-like features, convolution kernels, defocus test, Ultrascale+ MP SoC

## Abstract

One of the main challenges faced by iris recognition systems is to be able to work with people in motion, where the sensor is at an increasing distance (more than 1 m) from the person. The ultimate goal is to make the system less and less intrusive and require less cooperation from the person. When this scenario is implemented using a single static sensor, it will be necessary for the sensor to have a wide field of view and for the system to process a large number of frames per second (fps). In such a scenario, many of the captured eye images will not have adequate quality (contrast or resolution). This paper describes the implementation in an MPSoC (multiprocessor system-on-chip) of an eye image detection system that integrates, in the programmable logic (PL) part, a functional block to evaluate the level of defocus blur of the captured images. In this way, the system will be able to discard images that do not have the required focus quality in the subsequent processing steps. The proposals were successfully designed using Vitis High Level Synthesis (VHLS) and integrated into an eye detection framework capable of processing over 57 fps working with a 16 Mpixel sensor. Using, for validation, an extended version of the CASIA-Iris-distance V4 database, the experimental evaluation shows that the proposed framework is able to successfully discard unfocused eye images. But what is more relevant is that, in a real implementation, this proposal allows discarding up to 97% of out-of-focus eye images, which will not have to be processed by the segmentation and normalised iris pattern extraction blocks.

## 1. Introduction

Biometric identification by iris recognition is based on the analysis of the iris pattern using mathematical techniques. Although it is a relatively recent technique (the first automatic identification system was developed and patented by John Daugman in the last decade of the 20th century), its excellent identification characteristics have led to its rapid evolution. Thus, using the most recent developments, it has become a mature technique. The challenge now is to use it in a scenario where the cooperation of the person is not required to obtain a focused image of the eye but where the person can be allowed to continue walking, keeping the image sensor at a distance of more than 1 m from the person’s face. In iris recognition at a distance (IAAD) systems [1], it is common to use a camera that, thanks to its large field of view (FoV), detects and tracks the face of people approaching the system, and to have several high-resolution iris cameras, with a narrower FoV, that move according to what is determined by the first camera, in order to capture the image that will have the irises to be processed. These systems will therefore employ pan–tilt and control units. As the person is moving, capturing an image of the iris with the appropriate quality in contrast and resolution will require predicting where the person’s face will be at each instant to capture a quality image [2]. However, in cases where the field of view to be covered is not too large, such as an access controlled point, a static system, in which a single high-resolution camera is located, can be used. In this situation, the system shall be able to process these high-resolution images at high speed [3]. The need to capture high-resolution images is imposed by the maintenance of a certain FoV, which would otherwise be too low. On the other hand, having to capture a large number of frames per second is due to the fact that, under normal circumstances, the depth of field of the camera (i.e., the distance around the image plane for which the image sensor is focused [4]) will be very shallow [1]. Because the person to be identified is moving, it is difficult to time the camera shutter release to coincide with the moment when the iris is in the depth of field and therefore in focus. Only by processing many images per second can the system try to ensure that one of the images has captured this moment.

Capturing and pre-processing a large volume of input images requires the use of a powerful edge-computing device. Currently, the options are mainly in the form of graphics processing units (GPUs), application-specific integrated circuits (ASICs), or field-programmable gate arrays (FPGAs). Due to the high power consumption and size of GPUs, and the low flexibility of ASICs, FPGAs are often the most interesting option [5,6]. Moreover, if the traditional development approach of FPGAs using low-level hardware languages (such as Verilog and VHDL) is usually time-consuming and very inefficient, the use of high-level language synthesis (HLS) tools allows developers to program hardware solutions using C/C++ and OpenCL. This significantly improves the efficiency in FPGA developments [7,8]. Finally, FPGAs are nowadays integrated in multi-processor system-on-chips (MPSoC), in which computer and embedded logic elements are combined. These MPSoCs thus offer the acceleration capabilities of the FPGA and the computational capabilities that allow it to work as an independent stand-alone system, which does not have to be connected to an external computer/controller.

Using a MPSoC as the hardware basis (the AMD/Xilinx Zynq${}^{\mathrm{TM}}$ UltraScale+${}^{\mathrm{TM}}$ XCZU4EV), we recently proposed a real-time eye detection system, which was able to process the 47 frames per second (fps) provided by a EMERALD 16MP image sensor from Teledyne e2v [3]. In the actual deployment of this system, the eye images (640 × 480 pixels in size) were sent to an Intel i9 computer for processing and final identification of the user. The problem is that, as there is about 2–3 s of recording per user in which eyes are detected, the number of eye images sent to the external computer can exceed 250 images. The external computer cannot process this volume of information before the user has left the access point at a normal pace. The solution to this problem is to filter out the large number of images in which the iris is not in focus. In our case, this means discarding almost 97% of the eye images captured by the system.

The main contribution of this work is to describe the implementation, in the programmable logic (PL) part of the MPSoC, of a module that evaluates which points of an image are in focus. Integrated together with an eye image detection system, this module allows to discard the detections that do not pass this out-of-focus test, thus preventing them from being processed by the next stages of an iris recognition identification system. The whole framework was mainly built using Vitis HLS and synthesised in the aforementioned AMD/Xilinx UltraScale+ MPSoC (multiprocessor system-on-chip). Given the characteristics of an FPGA, the design option selected for this defocus blur evaluation module was based on convolution kernels [7,9].

The rest of the paper is organised as follows: The state of the art in the topic is briefly revised in Section 2. Section 3 provides an overview of the whole proposed framework for eye detection implemented in the processing system (PS) and programmable logic (PL) parts of the MPSoC and details about the implementation of the defocus estimation core, synthesised as a functional block in the PL of the MPSoC. Experimental results are presented in Section 4. Finally, the conclusions and future work are drawn in Section 5.

## 2. Related Work

Defocus blur is the result of an out-of-focus optical imaging system [4]. When an object is not in the focal plane of the camera, the rays associated with it do not converge on the same spot on the sensor but to a region called the circle of confusion (CoC). The CoC can be characterised using a point spread function (PSF), such as Gaussian, defined by a radius/scale parameter [10]. This radius increases as this object gets farther away from the focal plane. In practice, it is assumed that there is a range of distances from the camera, associated with the focal plane, at which an object is considered to be in focus. This is the so-called depth of field of the camera.

The detection of blurred image regions is a relevant task in computer vision. Significantly, defocus blur is considered by several authors to be one of the main sources of degradation in the quality of iris images [2,11,12,13,14]. Many single-image defocus blur estimation approaches have been proposed [4,10]. They can be roughly classified into two categories: edge-based and region-based approaches [10]. The edge-based approach models the blurry edges to estimate a sparse defocus map. Then, the blur information at the edge points can be propagated to the rest of the image to provide a dense blur map [15]. Edge blur estimation models typically consider that the radius of the CoC is roughly constant, and define the edge model using this parameter [16,17]. However, other more complex models of defocused edges can be used [18]. Although edge detection and blur estimation can be performed simultaneously [10], the problem with these approaches is that obtaining the dense defocus map can be a time-consuming step. To alleviate this problem, Chen et al. [16] proposed to divide the image into superpixels, and consider the level of defocus blur in them to be uniform. The method needs a first step in which this division into superpixels is generated. One additional problem with edge-based defocus map estimation is that they usually suffer from textures of the input image [19]. It should be noted that our region of interest, the iris, is primarily a texture region.

Region-based approaches avoid the propagation procedure to obtain dense defocus maps in edge-based approaches, dividing up the image into patches and providing local defocus blur estimation values. They are free of textures [19]. Some of these approaches work in the frequency domain, as the defocus blur has a frequency response with a known parametric form [20]. Oliveira et al. [21] proposed to assume that the power spectrum of the blurred images is approximately isotropic, with a power-law decay with the spatial frequency. A circular Radon transform was designed to estimate the defocus amount. Zhu et al. [22] proposed to measure the probability of the local defocus scale in the continuous domain, analysing the Fourier spectrum and taking into consideration the smoothness and colour edge information. In the proposal by Ma et al. [23], the power of the high, middle and low frequencies of the Fourier transform is used. Briefly, the ratio of the middle-frequency power to the other frequency powers is estimated. This ratio should be larger for the clear images than for the defocused and motion blurred images. For classifying the images into valid or invalid ones, a support vector machine (SVM) approach is used. In all cases, the approach is simple and fast. These approaches take advantage of the fact that convolution corresponds to a product in the Fourier domain. Also assuming spatially invariant defocus blur, Yan et al. [24] proposed a general regression neural network (GRNN) for defocus blur estimation.

To avoid the computation of the Fourier transform, other researchers prefer to directly work in the image domain. As J.G. Daugman pointed out [25], defocus can be represented, in the image domain, as the convolution of an in-focus image with a PSF of the defocused optics. For simplicity, this function can be modelled as an isotropic Gaussian one, its width being proportional to the degree of defocus [21]. Then, in the Fourier domain, this convolution can be represented as

(1) $$D_{\sigma }\left(\mu ,\nu \right)=e^{-\left(\frac{\mu^{2}+\nu^{2}}{\sigma^{2}}\right)}F\left(\mu ,\nu \right)$$

**Figure 1:**
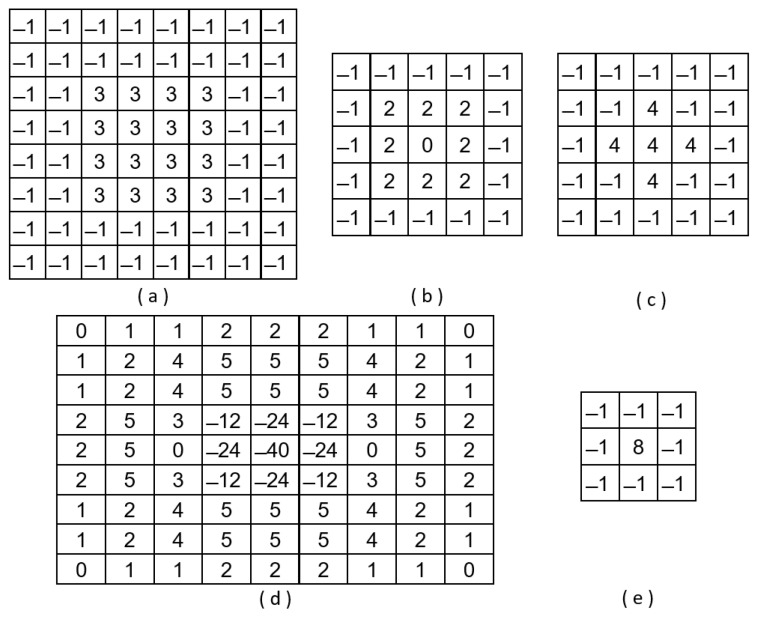
Convolution kernels proposed by (**a**) Daugman [25], (**b**) Wei et al. [11], (**c**) Kang and Park [29] and (**d**,**e**) Wan et al. [30].

$D_{\sigma }\left(\mu ,\nu \right)$ and F(μ,ν) are the 2D Fourier transforms of the image defocused to degree σ and the in-focus image, respectively. Significantly, for low (μ,ν) values, the exponential term approaches unity, and both Fourier transforms are very similar. The effect of defocus is mainly to attenuate the highest frequencies in the image [25]. As the computational complexity of estimating the Fourier transform is relatively high, Daugman suggested to take into consideration Parseval’s theorem

(2) $${\;\int \int \;|I\left(x,y\right)|}^{2}dxdy=\;\int \int \;{\left|F\left(\mu ,\nu \right)\right|}^{2}d\mu d\nu$$

and to not estimate the total power at high frequencies in the Fourier domain but in the image domain. Thus, the idea is to filter the image with a high pass (or a band-pass filtering within a ring of high spatial frequency). After filtering the low-frequency part of the image, the total power in the filtered image is computed using the equation

(3) $$P=\frac{1}{M\cdot N}\sum_{i=0}^{M-1}\sum_{j=0}^{N-1}{\left|C\left(i,j\right)\right|}^{2}$$

where C(i,j) is the filtered image of M×N dimension. In order to reduce the computational complexity of the Fourier transform, Daugman proposed to obtain the high frequency of the image using a 8 × 8 convolution kernel (Figure 1a). Briefly, this kernel is equivalent to superposing two centred square box functions with a size of 8 × 8 (and amplitude −1) and 4 × 4 (and amplitude +4). The 2D Fourier transform of this kernel can be expressed as the equation

(4) $$K\left(\mu ,\nu \right)=\frac{sin(\mu )sin(\nu )}{\pi^{2}\mu \nu }-\frac{sin(2\mu )sin(2\nu )}{4\pi^{2}\mu \nu }$$

**Figure 2:**
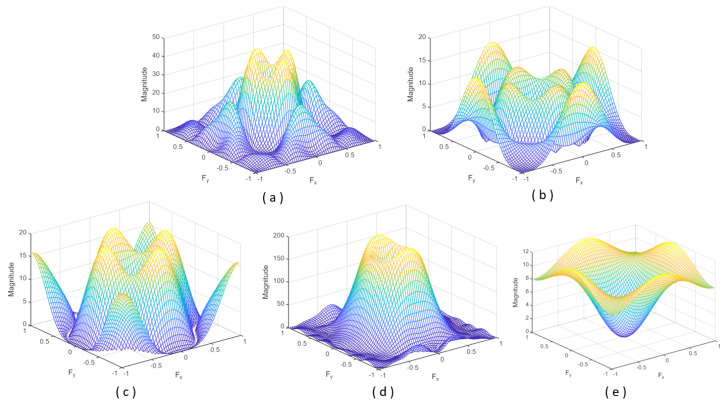
Fourier spectrum for the convolution kernels (Figure 1) proposed by (**a**) Daugman [25], (**b**) Wei et al. [11], (**c**) Kang and Park [29], and (**d**,**e**) Wan et al. [30].

In short, the result is a band-pass filter with a central frequency close to 0.28125 and with a bandwidth of 0.1875. The Fourier spectrum of this kernel is shown in Figure 2a.

It must be noted that, although there is no reference image, to obtain a normalised score between 0 and 100, Daugman proposed that the obtained spectral power *x* be passed through a compressive non-linearity of the form

(5) $$f\left(x\right)=100\times \frac{x^{2}}{x^{2}+c^{2}}$$

where *c* is the half power of a focus score corresponding to 50%. This last normalisation step presupposes the existence of a canonical (reference) iris image [26].

Finally, once the signal power value associated with the image (or a sub-image within the image) has been obtained, a threshold value can be set for determining whether the image is clear or out-of-focus [14].

Since, in our scenario, it is possible to assume the isotropic behaviour of the PSF and that the blur is due to bad focusing, this approach based on convolution kernels applied in the image domain is fully valid [21,25]. Furthermore, the convolution filtering of digital images can be efficiently addressed using FPGA devices [7,27,28].

Similar to Daugman’s filter, the proposal by Wei et al. [11] is a band-pass filter, but it selects higher frequencies (central frequency around 0.4375 and a bandwidth of 0.3125). The convolution kernel is shown in Figure 1b. It is a 5 × 5 kernel that superposes three centred square box functions. The frequency response is shown in Figure 2b. Kang and Park [29] also proposed a kernel with a size of 5 × 5 pixels:

(6) $$K\left(\mu ,\nu \right)=\frac{sin\left(\frac{3}{2}\mu \right)sin\left(\frac{3}{2}\nu \right)}{\frac{9}{4}\pi^{2}\mu \nu }-\frac{sin\left(\frac{5}{2}\mu \right)sin\left(\frac{5}{2}\nu \right)}{\frac{25}{4}\pi^{2}\mu \nu }-4\cdot \frac{sin\left(\frac{1}{2}\mu \right)sin\left(\frac{1}{2}\nu \right)}{\frac{1}{4}\pi^{2}\mu \nu }$$

This band-pass filter has a central frequency close to 0.2144 and a bandwidth of 0.6076. Thus, the shape of the Fourier spectrum is similar to the one of Daugman’s proposal but with a significantly higher bandwidth (see Figure 2c). Figure 1c shows that it combines three square box functions (one of size 5 × 5 and amplitude −1, one of size 3 × 3 and amplitude +5, and other of size 1 × 1 and amplitude −5).

Since high frequency is associated with sharp changes in intensity, one way to estimate its presence in the image would be to use the Laplacian [30]. The Laplacian L(x,y) of an image I(x,y) is given by

(7) $$L\left(x,y\right)=\frac{\partial^{2}I}{\partial x^{2}}+\frac{\partial^{2}I}{\partial y^{2}}$$

This operator is the basis for the convolution kernel proposed by Wan et al. [30]. To reduce the sensitivity to noise, the Laplacian is applied to an image that is first smoothed by a Gaussian smooth filter. The 2D LoG (Laplacian-of-Gaussian) function has the form

(8) $$LoG\left(x,y\right)=-\frac{1}{\pi \sigma^{4}}\left[1-\frac{x^{2}+y^{2}}{2\sigma^{2}}\right]e^{\frac{x^{2}+y^{2}}{2\sigma^{2}}}$$

with σ being the Gaussian standard deviation. For σ equal to 1.4, the convolution kernel takes the form shown in Figure 1d. The associated Fourier spectrum is illustrated in Figure 2d. However, to simplify the computation, the authors propose an alternative 3 × 3 Laplace operator, combining two square box functions (one of size 3 × 3 and amplitude −1, and other of size 1 × 1 and amplitude +9) (Figure 1e).

## 3. Implementation

### 3.1. Overview of the Proposed Framework

**Figure 3:**
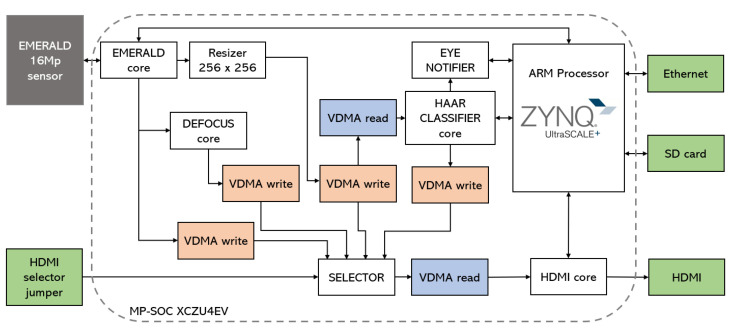
Overview of the proposed framework.

Figure 3 shows the schematic of the proposed logic architecture for detecting iris images, in which the core for evaluating defocus is integrated. The figure shows how the images are captured from a Teledyne e2v EMERALD sensor. This sensor can provide 16 Mpx images at a speed of 47 fps, using 16 low voltage differential signalling (LVDS) lines. The first of the cores in the architecture, EMERALD core, is responsible for deserialising the signals encoded on these 16 LVDS lines, generating the input video stream. A first video direct memory access (VDMA) channel allows up to 8 frames to be stored in the DDR3 RAM available on the hardware platform.

The size of the frames received in the input video stream is modified by the Resizer core to a size of 128 × 128, which is also stored in the RAM using a second VDMA channel. In addition, the input stream is processed by the DEFOCUS core to generate a binary image stream, in which out-of-focus areas are marked with 0 and in-focus areas with 1. These images are stored in RAM using a third VDMA channel. The algorithm implemented in the DEFOCUS core is discussed in detail in Section 3.2.

The detection of the eyes present in the rescaled image is carried out by the HAAR CLASSIFIER core. The core reads from the VDMA that manages this stream of rescaled images and implements a parallelised version of the popular classifier proposed by Viola and Jones [31]. Briefly, this detector uses a set of Haar-like features to characterise an image region and a supervised learning scheme (AdaBoost) to boost the classification performance of a simple learning algorithm. The result is an ensemble of weak classifiers, each of which internally computes a Haar-like feature and uses a threshold value to determine whether the region can be the desired object or not. As described in [3], the classifier is not organised as a sequence of weak classifiers but as a decision tree [32]. Thus, instead of having to use hundreds of classifiers (which will have to be evaluated almost entirely if the region to be studied has similarity to the person’s eye), the system evaluates 120 Haar features in parallel, which form five stages, each of which has three trees with 8 nodes each (5 × 3 × 8 features) [3]. All five stages are executed in parallel.

**Figure 4:**
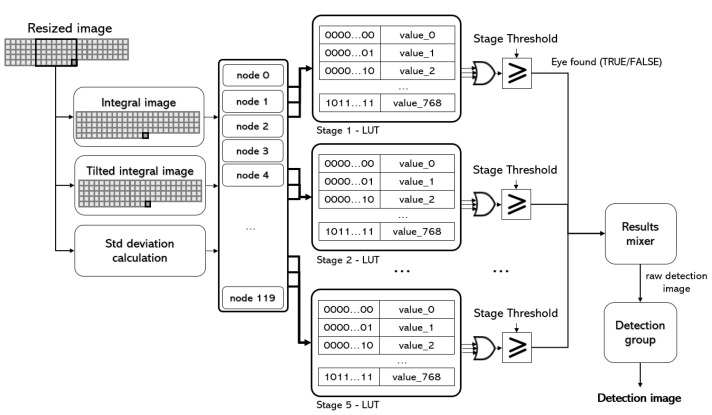
The classification structure.

**Figure 5:**
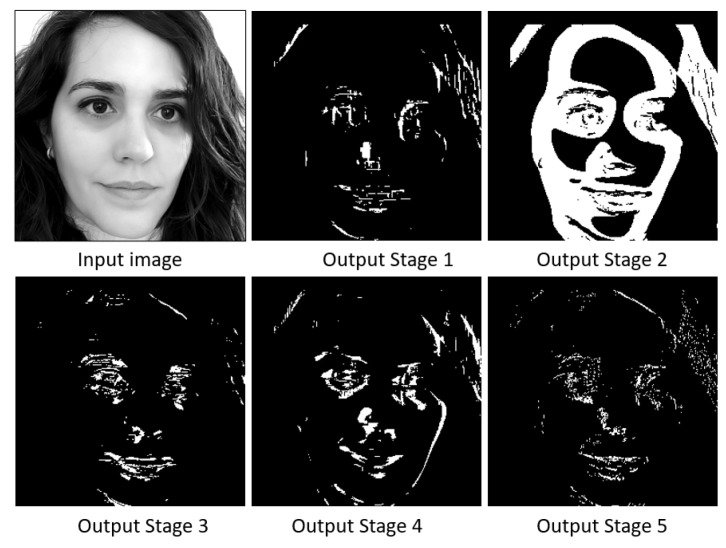
Input image and outputs provided by the five stages in the classifier.

**Figure 6:**
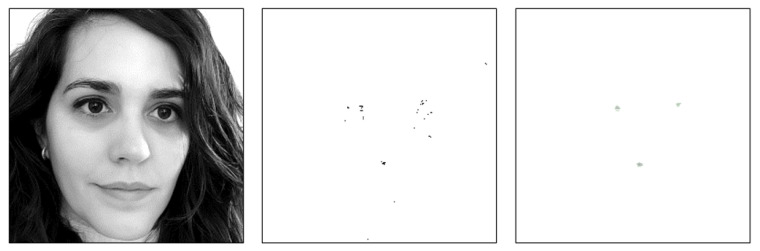
(**Left**) Input image; (**middle**) raw detection image; and (**right**) filtered detection image. Both images are inverted in colour to help visualise the points obtained.

Figure 4 shows the internal scheme of the classifier. First, the integral image, the tilted integral image, and the standard deviation of the grey level of the image are calculated [31,32]. These parameters allow the parallel evaluation of the 120 Haar features which, compared to a threshold, generate 120 binary values (node 0 to 119). With these values, 15 vectors of 10 components are formed. In each vector, the first two bits encode the tree within the stage, and the next eight are taken from the evaluation of eight nodes. The vectors are grouped in threes, and each group of three vectors is used to address a look-up table (LUT). This allows three values per LUT to be obtained, which are summed. Theoretically, the final value of each tree should be computed by multiplying each node by a weight and summing the results. In order to accelerate the execution, the LUT is implemented with the results of each 256 possibilities. This allows us to remove the hardware employed for math computations and have the results in a single clock. Finally, the resulting value is compared to a threshold. This comparison generates a Boolean value, which determines whether that stage evaluates the region as an eye or not. Figure 5 provides the output images from the five stages in the classifier for a given input image. If the output Boolean values computed by the five stages are true, the evaluated region is marked as true (results mixer core). Figure 6 (middle) shows the raw detection image associated to the five output images in Figure 5. Significantly, the whole process is executed in only four steps [3]. The raw detection image, the output of the results mixer core, is slightly smaller than the original rescaled one (if the size of the rescaled image and evaluated region are M×N and m×n, respectively, the size of this image will be (M−m+1)×(N−n+1)).

The problem with this scheme is that the jump between the regions being evaluated is one pixel, so the overlap between regions is very large. This results in very close positive detections, which generates many images that are associated with a single iris image. The detection group core filters the raw detection image, adding the values within a sliding window and thresholding the obtained sum value to mark the pixel as a positive detection (its value will be equal to the sum value) or not (0). Figure 6 shows the raw detection image associated to an input image, and the filtered version obtained by the detection group core. The sliding window is 20 × 15 pixels, and the threshold value is set to 7 (it can be modified if desired). Both images in Figure 6 are inverted in colour to help visualise the points obtained. It can be noted how the detection group core filters the isolated dots of the raw detection image, and groups the dots into higher entities. In fact, three major entities are identified (both eyes and one false positive detection).

The eye notifier core is responsible for providing high-resolution image cropping (640 × 480) for each entity detected in the image. A sliding window is now used to detect the maximum values inside the entities provided by the detection group core. For each entity, a 640 × 480 pixel image is cropped from the high-resolution image. This cropping is centred on the position, transposed from the scaled image to the original input image, provided by the maximum detected value.

### 3.2. Defocus Estimation

The basis of the designed defocus blur estimation core is convolution. A 2D convolution can be mathematically represented by the equation

(9) $$C\left(i,j\right)=\sum_{u=-U}^{U}\sum_{v=-V}^{V}h\left(u,v\right)\cdot I\left(i-u,j-v\right)$$

with I(i,j) and C(i,j) being the input and the filtered images, respectively. h(u,v) is the convolution kernel, with size (2·U+1)×(2·V+1). If the size of the convolution kernel is 8×8, the expansion of Equation (9) results in 64 multiplications and 63 summations to be computed for each pixel.

If a large storage capacity is available (as is the case when using CPU or GPU), it is possible to store the complete rows read from the image sensor, and apply convolution when the complete dataset is available. This is not the situation when working with an FPGA. If the input image and convolution kernel are small in value (e.g., Wei et al. [7] work with 640 × 480 pixel images and a convolution kernel of 3 × 3), FIFO (first in–first out) memories can be implemented to store the necessary image rows (3 FIFO memories with a depth of 637 each in the case of Wei et al.’s implementation). In our case, the EMERALD 16MP sensor provides images of 4096 × 4096 pixels, and the design will need 8 FIFO memories with a depth of 4088 (4096 − 8) each. Storing these data in order to apply the convolution kernel when all the data are available would increase the data usage and latency.

**Figure 7:**
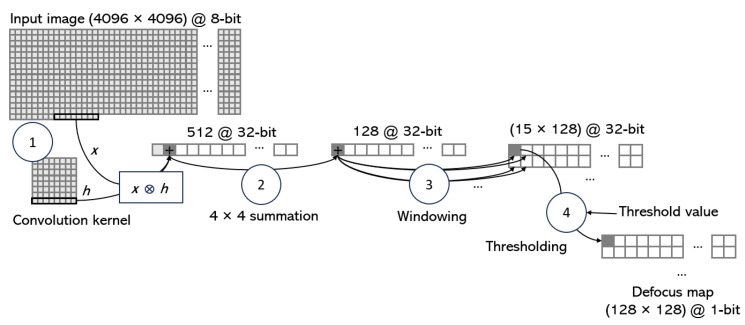
Graphical representation of the defocus blur map estimation. The first step implies the generation of a line buffer of 512 positions with 32-bit values. Each position in this buffer stores the convolution of a 8 × 8 block of the input image with the convolution kernel. The second step is a 4 × 4 summation for generating a line buffer of 128 positions with 32-bit values. A sliding window of 20 × 15 size is used in this map to accumulate the values (Step 3). A final thresholding process permits the core to obtain the final defocus map.

However, it is important to note that, in our case, the defocus map obtained must evaluate the pixels of the detection image (the output of the eye notifier core, see Figure 4). The size of this detection image is much smaller than that of the input image. Therefore, an initial data reduction step is to apply the convolution kernel not to each pixel of the input image but in steps of *S* pixels as proposed by Daugman [25]. In our case, as the sensor data are read in blocks of 8 pixels, a value of *S* equal to 8 is used. Applying the convolution kernel in 8 pixel steps allows, in one clock cycle, to have the 8 multiplications and 7 additions of that kernel row. Using a memory of 4096/8·32 bits, it is possible to store, without losing resolution, the summation and to accumulate results until the results of the last row of the kernel are obtained. The result is a defocus map of size 512 × 512, storing 32-bit values. Given the size of the scaled image (128 × 128), we further reduce the size of the defocus map by adding in 4 × 4 blocks and obtaining a 128 × 128 map, with 32-bit values. Figure 7 schematizes the procedure for obtaining the defocus map. The first and second steps involve convolving the kernel with the input data (in batches of 8 pixels per clock) and performing 4 × 4 compression. Both steps require only two line buffers: one of 512 and one of 128 values (i.e., whole images are not managed). The data are stored as 32-bit values.

The third step averages the defocus map using the same sliding window used to detect eyes (of size 20 × 15 in our implementation). Actually, to reduce computations, the values in the window are not averaged but simply accumulated. The result obtained is thresholded using a configurable value from the ARM (step 4 in Figure 7). In this way, the final result is a defocus map with the same size as the detection image obtained by the classifier and which stores binary values (0 if the value is not in focus and 1 otherwise). Using a VDMA channel, the map is stored to be used for validating each positive eye detection.

In order to speed up the design of this core, as well as to optimise its performance characteristics, the Vitis HLS tool (https://docs.xilinx.com/r/en-US/ug1399-vitis-hls/Introduction (accessed on 15 August 2023)) from Xilinx is used. Vitis HLS allows the developer not to have to generate RTL using a hardware HDL language but to use a high/medium level language (C/C++, System C) and obtain, from these sources, the core IP in RTL. In our case, the design is coded using C/C++. From the set of directives that Vitis HLS provides to help developers optimise a hardware design, we implement the PIPELINE directive to parallelise the execution of the multiple computations in the convolution operation. In addition, in step 4 (thresholding), the UNROLL directive is used to transform loops by creating multiple copies of the loop body in the RTL design. In this way, some or all iterations of the loop can occur in parallel. Finally, we use HLS directives to establish when Block RAMs (BRAMs) or UltraRAM (URAMs) should be used in the design. BRAMs are required to be dual ported. URAM blocks have a fixed width of 72 bits, so two 32-bit values are joined together to be stored at each location.

The convolution kernels described in Section 2 are implemented and tested in the DEFOCUS core of the proposed framework. Table 1 shows the resource usage for the DEFOCUS core implementing Daugman’s proposal. As a detail, regarding memory, everything is optimised to fit in one URAM and two BRAM, as the rest of the design demands a lot of BRAM (which is faster but has been used for the HAAR CLASSIFIER). The total resource usage for the whole system is shown in Table 2.

## 4. Experimental Evaluation

### 4.1. Experimental Setting

**Figure 8:**
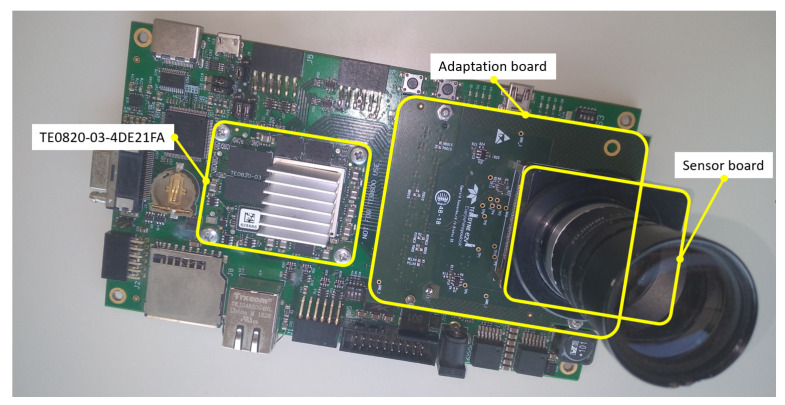
The first version of the system, mounting the TE0820-03-4DE21FA and the adaptation board on a carrier board for Trenz Electronic 7 Series. The adaptation board in turn mounts the sensor board (with image sensor and optics).

The system is built as a portable device. The computational core is the TE0820-03-4DE21FA micromodule from Trenz Electronic. This micromodule is an industrial-grade 4 × 5 cm MPSoC System on Module (SoM) integrating an AMD/Xilinx Zynq${}^{\mathrm{TM}}$ UltraScale+${}^{\mathrm{TM}}$ XCZU4EV. Moreover, the micromodule includes 2 GByte DDR4 SDRAM, 128 MByte Flash memory for configuration and operation, and powerful switch-mode power supplies for all on-board voltages. A large number of configurable I/Os is provided via rugged high-speed stacking connections. The TE0820-03-4DE21FA micromodule is mounted on a compatible carrier board that provides physical connections between the module and peripherals. On this first version of the hardware system, the carrier board for the Trenz Electronic 7 Series was used. This carrier provides us with all the components needed during the development phase, such as power delivery, Ethernet connection, debugging interface, UART to USB bridge, HDMI, PMOD connectors, and a FMC (FPGA Mezzanine Card) connector. The image sensor is the EMERALD 16MP from Teledyne e2v, which is mounted on a sensor board. Apart from other I/O connections, the sensor board provides as outputs the LVDS lines. An adaptation board was designed to mount the sensor board and to interface with the FMC connector in the carrier board. Figure 8 provides a snapshot of this first version of the camera.

**Figure 9:**
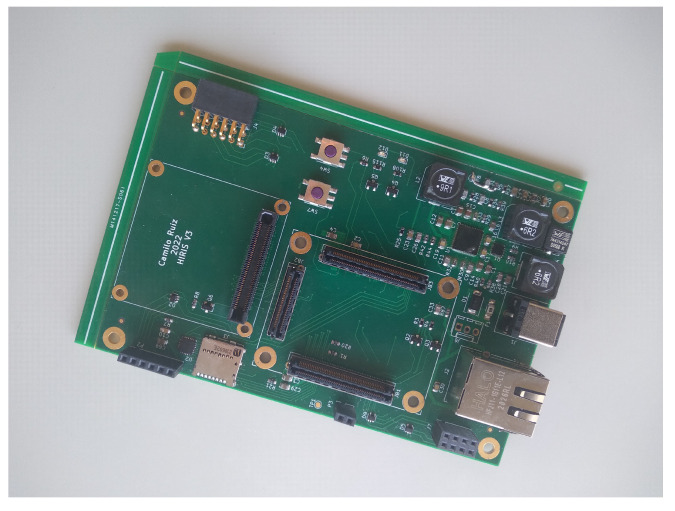
Custom PCB design of the carrier board.

**Figure 10:**
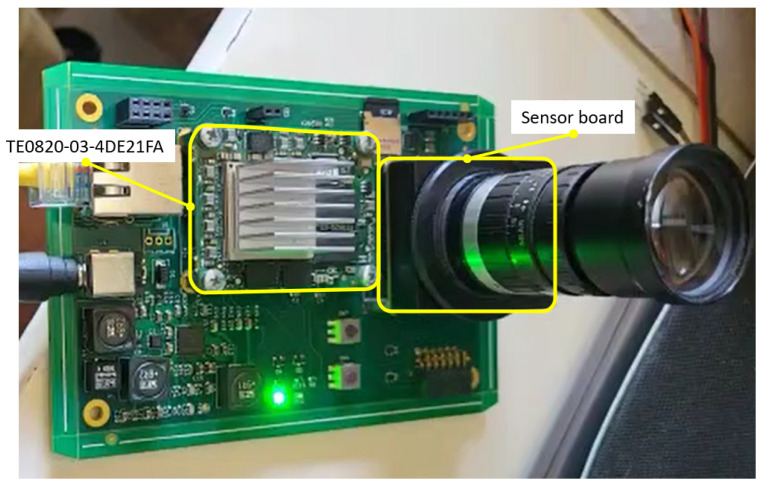
The final version of the system, mounting the TE0820-03-4DE21FA and the sensor board on a customised carrier board.

The carrier board for Trenz Electronic 7 Series provides peripherals that are not strictly necessary. In order to customize this carrier board, and also to remove the adaptation board from the scheme, a specific carrier board was designed and tested. Thus, in this final version, the carrier board includes only the peripherals needed by the proposed system. Parts that were initially used for debugging (HDMI, debugging interface, and UART USB bridge) were also left out. This resulted in an smaller and cheaper board (see Figure 9 and Figure 10).

**Figure 11:**
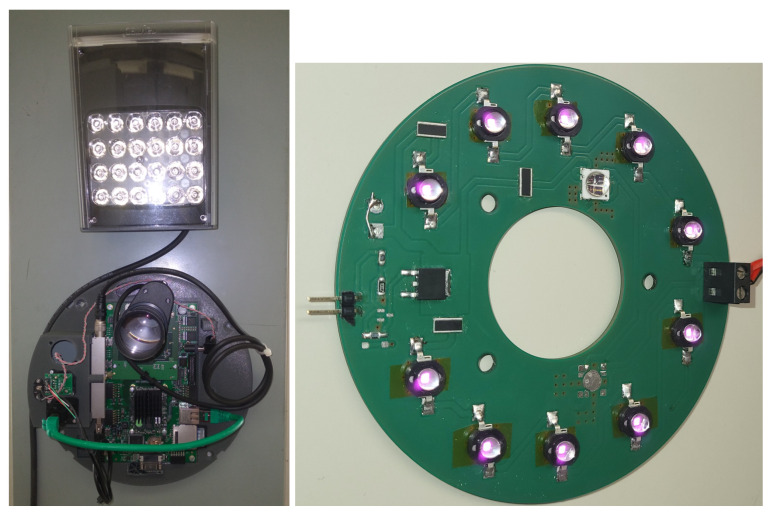
(**left**) The previous version of the hardware system, showing the VARIO2 IPPoE infrared lamp from Raytec, and (**right**) the board design of the new illumination module.

With respect to the illumination subsystem, the EMERALD 16MP features a very small true global shutter pixel (2.8 µm). Moreover, the sensor was designed to exhibit a very reduced dark signal nonuniformity (DSNU) value. Both properties allow the sensor to correctly work in a low-light scenario. In any case, the first hardware design employs a VARIO2 IPPoE infrared lamp from Raytec (see Figure 11 (left)). Similar to the proposal of Dong et al. [33], this lamp cannot be synchronised with the trigger of the camera and provides a very wide angle when compared with the FoV covered by the EMERALD sensor.

The Raytec lamp provides 51W continuously. To avoid this continuous irradiation, a plate with high power LEDs (3W) was designed and synchronised with the triggering of the camera. The board design is shown in Figure 11 (right). In total, 10 LEDs are mounted on the board. The new system ensures sufficient brightness and allows shortening the exposure time of the sensor, reducing motion blur caused by the subject walking through the FoV of the camera. The new design also ensures a more homogeneous illumination of the person’s face in the capture position (about 1.7 m from the camera).

### 4.2. Hardware Implementation

In the design of the cores that are integrated in the programmable logic (PL) region of the AMD/Xilinx device, intensive use is made of Vitis High-Level Synthesis (HLS). Specifically, the two main streams described in this manuscript are synthesised in two cores generated from C++ files using HLS. The first one includes all the algorithms necessary to detect the eye regions: calculation of the integral and tilted integral images, calculation of the standard deviation, image processing using the HAAR features in the five stages described, and the subsequent filtering that generates the dot image with the positive detections. The second includes the focused region detection algorithm. Synthesising and merging all modules into only two cores allows the employed synthesis and implementation tool to further optimize and share resources. To achieve a faster runtime and allow the tool to optimise resources, source C++ files are subsequently modified by adding specific directives (see Section 3.2).

In the proposed design, all the hardware implemented on the programmable logic (PL) part is initialised and controlled from the processing system (PS) part (in this case, in the Cortex-A53 ARM processor available in the SoC). Initialisation follows a series of stages. First of all, the peripherals are tested. Then, the EMERALD sensor is initialised. After that, the onboard memory is configured and synchronised with the VDMA cores. It can be noted that there are four VDMA write cores (see Figure 3). These video streams (input images, resized images, contrast images, and detection images) can be viewed in real time using the HDMI interface. This allows easy real-time debugging. Finally, the EMERALD sensor is configured to produce 47 fps at 16MP, and the video stream is started. Alternatively, for debugging purposes, the video stream can be generated from a still image stored on an SD card.

When the desired object (an eye) is found on the video stream, the eye notifier interrupts the ARM processor and saves the frame number and coordinates. When the processor attends to the interrupt, it reads the data, checks the defocus map to determine if the detection is in focus, and, if so, it is considered a valid detection. If the detection is a valid one, the processor goes to the referenced frame temporary stored on the frame buffer used by the VDMA, crops the region, and stores it into another buffer that will be sent through Ethernet. By dividing the tasks between the two ARM cores, the system can send the detected eyes (in 640 × 480 images) with minimal detection delay (variable depending on the number of eyes detected per input image).

The processing is made around a video stream implemented using an AXI Stream interface. As a throughput of at least 47 fps at 16MP resolution is required (to get the most out of the image sensor), the design choice was to use a data width of 8 bytes per clock at 150 MHz. As described in Section 3.1, this video stream is buffered in the onboard DDR3 memory by means of VDMA cores. This allows the ARM processor to access the frames to accomplish the cropping and sending steps. The hardware resource usage is shown in Table 2. The intensive use of block RAMs (BRAMs) in the classifier module is due to the need to store pixels already read from the sensor but needed to estimate the integral and rotated integral images, and to calculate the standard deviation (see Figure 4). It is also due to the storage needs of the result mixer and detection group modules. They are then used to implement memories and FIFOs. One UltraRAM (URAM) is employed in the defocus module. The DSP48 blocks are intensively used for implementing the arithmetic required in the classifier module.

### 4.3. Obtained Results

The set of tests carried out to check the validity of the proposal is divided into three blocks. In the first, a publicly available database, the Clarkson dataset LivDet2013 [34], is used to evaluate the ability of the convolution kernel as an estimator of the level of defocus blur. This database includes sets of images with different levels of defocus blur. They are images of eyes, so they do not allow evaluation of the eye detection system. In the second test block, the CASIA-Iris-Distance V4 database (http://biometrics.idealtest.org/ (accessed on 15 August 2023)) is extended by incorporating slightly blurred versions of the component images. In this case, it is possible to evaluate the system’s ability to detect eyes and to discard those detections that are out of focus. Finally, a third block of tests is carried out in a real environment, using the hardware described above. In addition to evaluating the system’s ability to detect eyes in a real environment (using the EMERALD 16MP sensor and with people in motion), these tests allow us to determine that the system is capable of discarding a large volume of out-of-focus detections. The following sections provide details on these three test blocks.

#### 4.3.1. Evaluation of the Defocus Blur Estimation in Eye Subimages

Although the actual validation of the proposal must be performed with the system described in Section 4.1, in order to evaluate the performance for estimating the iris image defocus of the four convolution kernels described in Section 3.2, the Clarkson dataset LivDet2013 [34] is used. In this dataset, images are collected through the use of video capture of 100 frames at 25 fps using a Dalsa camera. The sequence is started out of focus and is moved through the focus range across full focus and back to being out of focus. Images are grouped according to their blur level in five categories:

- Group #1–10% blur in frame before least blurry image;
- Group #2–5% blur in frame before least blurry image;
- Group #3 least blurry image;
- Group #4–5% blur in frame after least blurry image;
- Group #5–10% blur in frame after least blurry image.

**Figure 12:**
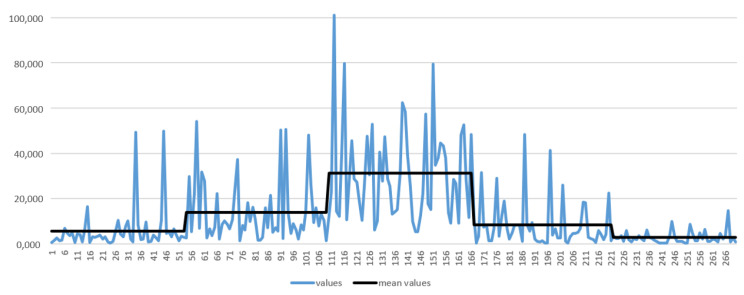
Mean filter responses (in blue colour) obtained from applying Daugman’s [25] filtering to the 270 images in the training set of the Clarkson dataset LivDet2013 [34]. The five steps that form the black line are associated with the five categories in the database. The value of each step is the average value of the responses obtained on the images in each category.

A total of 270 images are available for training. In Figure 12, the blue curve joins the response values for applying the convolution kernel proposed by Daugman [25] to these images. A higher value indicates that the image has, on average, a higher focus. The images are sorted by groups (the first 53 belong to group #1, the next 55 to group #2, and so on). The mean values of each group are shown in black (the curve forms five steps). The values cannot be clearly delimited into a range per group, as there are images with values much higher (or lower) than the group mean. The results obtained by applying the convolution kernels proposed by Wei et al. [11] and Wan et al. [30] are very similar, varying only mainly in the absolute value of the results.

**Figure 13:**
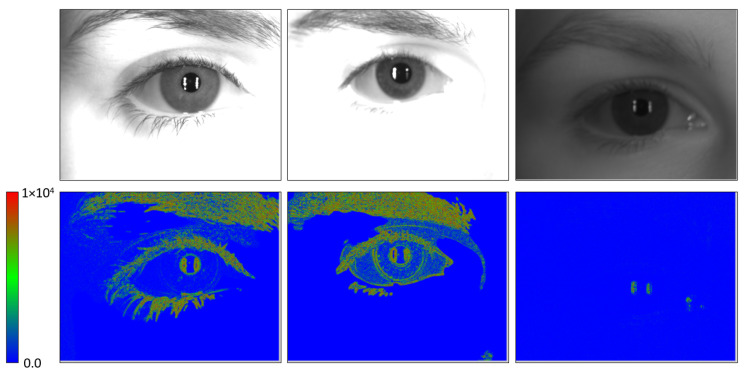
(**Top**) Examples of images with low contrast from the Clarkson dataset LivDet2013 [34]. The three images are included in the group #1 (10% blur in frame before least blurry image). (**Down**) Filter responses obtained when applied Kang and Park’s [29] filtering to the images in the top row. The scale values is shown at the left (responses greater than 1 $\times \;10^{4}$ are drawn in red colour).

The problem when evaluating an image is that the value averages those associated with each pixel. As shown in Figure 13, the contrast value of the image is not really the one associated with the iris. In this figure, the three images shown belong to group #1. However, while the first one has a very high average value (4201.04 using Kang and Park’s proposal), the third one has a very low value (92.55). In both cases, the iris area is clearly out of focus (blue values in the filter response images), but the presence of the eyebrows, in the first and also in the second image (978.43 average value), makes the average value high. Eyebrows and eyelashes offer higher values of focus if, in addition, the skin area of the face is saturated by the infrared illumination (for example, in the third image, where the eyebrow is also visible, the pixels associated with it are barely in focus).

Joining groups 1 and 5, and 2 and 4 (where the level of blur is the same), you have three groups (10% blur, 5% blur, and in focus). In Table 3, the results of the evaluated kernel approaches are summarised. The first column in the table identifies the kernel, and the rest of the columns provide the mean and standard deviations of the total power at high-frequency bands processing each kernel with the images in the three groups. For Wan et al.’s proposal, the 3 × 3 convolution kernel is used (Figure 1e). Given the dispersion shown in Figure 12, the Z-score (68% confidence interval) is employed to remove outliers before estimating the parameters for each group. The last column in the table illustrates the threshold values for the discrimination of defocus and in-focus images. ROC (receiver operation characteristic) curves are used to obtain these threshold values. Using these schemes, the 246 images available for testing in the Clarkson dataset LivDet2013 [34] are evaluated. Using the threshold values shown in Table 3, the number of rejected iris images (out-of-focus images) that are actually focused images (false rejection rate, FRR) is less than 1% for all tested kernels. This is because the problems present in the images mostly force an increase in the power obtained. This causes them to be falsely taken as in-focus images (increasing the false accept rate (FAR)). It is important to note that the FRR is the percentage of error valid for us, since what the system cannot do in any case is to discard any image with the iris that really is in focus.

#### 4.3.2. Quantitative Evaluation Using an Extended CASIA-Iris-Distance V4 Database

**Figure 14:**
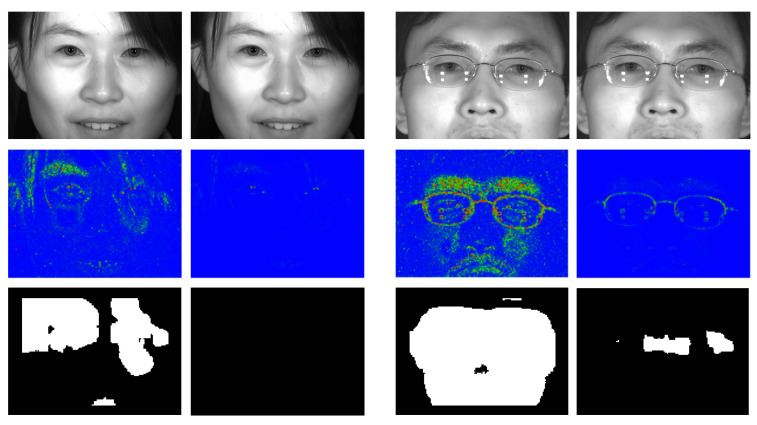
(**Top**) Original images from the CASIA-Iris-Distance V4 database and, on the right, defocused versions. (**Middle**) 147 × 108 pixel images obtained after applying the first two steps (see Figure 7) of the defocus blur estimation process. The blue tones are associated with out-of-focus pixels, while the green and red tones are associated with increasingly focused pixels. (**Bottom**) Defocus maps associated with the images in the top row. When the person wears glasses, these defocus maps are not completely zeroed, which causes certain eye detections to be falsely considered to be in focus (see text).

Significantly, when working with real images, the defocus test does not apply to eye images (such as those shown in Figure 13). In contrast, when the test is applied, there is no estimation of where the eye is, and what is done is to compute the convolution kernel values and threshold them using the previously obtained values. The kernel allows regions that are correctly focused (and have edges) to be marked with a value of 1 on a background value of 0. For obtaining quantitative results, the proposed approach is evaluated using an extended version of the CASIA-Iris-Distance, version 4.0 database. The original database contains 2567 images of 142 people, most of them graduate students of the Chinese Academy of Sciences’ Institute of Automation (CASIA). The database is captured indoors, with a distance of more than 2 m, and using a self-developed long-range multi-modal biometric image acquisition and recognition system (LMBS). In our extended version, the dataset is doubled, incorporating, for each image in the database, a version affected by defocus blur, simulated using a Gaussian of radius 2 pixels. The effect of this filtering is virtually unnoticeable. For example, Figure 14 shows two original database images and, to their right, the generated versions. Only by zooming in can the smoothing effect be seen.

The images have a size of 2352 × 1728 pixels. As in the real deployment, the convolution kernel is applied on a block of 8 × 8 pixels without overlapping, resulting in an image of 294 × 216 pixels. In our case, an array of 294 positions would be sufficient to store 32-bit data. The next step applies a compression that, instead of using a 4 × 4, uses a 2 × 2 block. Thus, the result is an image of 147 × 108. Although it would be sufficient to use an array of 147 positions, complete images are generated to show them as intermediate results in Figure 14 (middle). In the images, the defocus blur values are shown on a scale ranging from pure blue tone (out of focus) to green and, finally, red tones (higher level of focus). Using a sliding window of 23 × 17 pixels and a threshold value proportional to the ones in Table 3, the system provides the defocus maps shown in Figure 14 (bottom). It can be seen how the presence of glasses or reflections generates strong edges, which are not considered to be out of focus, and which generate defocus maps that are not completely zeroed.

The eye detection results in this database are 100%, matching those obtained when only the original version is used [3]. Of the total set of 10,268 eyes present in the entire extended database, 5016 images are correctly discarded for being out of focus. Of the total set of 10,268 eyes present in the entire extended database, 5016 images are correctly discarded for being out of focus. In total, 118 eye images are erroneously categorised as being in focus. All of them belong to images of people wearing glasses.

#### 4.3.3. Evaluation in a Real Scenario

**Figure 15:**
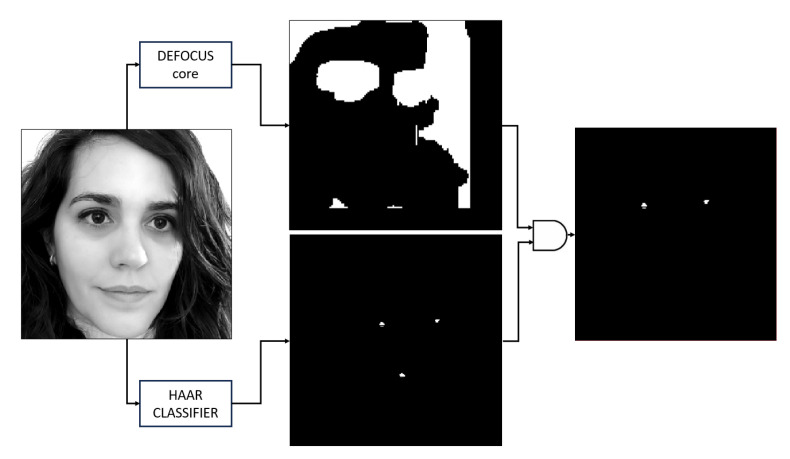
Generation of the contrast and eye detection masks from an input image, and combination for obtaining the final detection points. Both masks are of size 128 × 128 pixels.

The whole framework is tested in a real deployment. Figure 15 schematically illustrates how it works. Given an input frame, the framework simultaneously generates two masks. The upper image is generated by detecting the in-focus areas (in the example, they are marked in white colour; planar areas are also marked as defocus regions). The second mask is generated by the HAAR CLASSIFIER kernel. In this case, the white dots correspond to positive detection values. Both masks are merged by the ARM to generate a final mask that will be used to crop the eyes from the 16MP input image (white dots in the right image in Figure 15 (right)).

**Figure 16:**
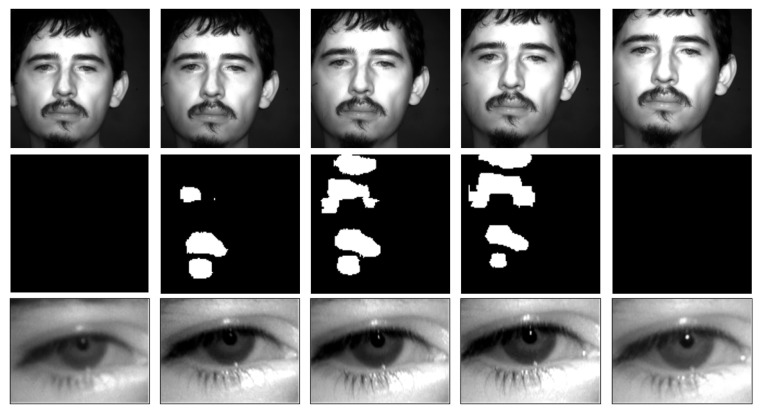
(**Top row**) Frames of a sequence of a person passing in front of the sensor. (**Middle row**) Masks obtained by the DEFOCUS core (white values are associated with regions in focus). (**Bottom row**) Images of the left eye of the images in the top row. It can be seen how the third and fourth images are in focus, while the rest are not.

Figure 16 shows an example of zooming in on the sensor. In the first frames on the left, the face is not in the depth of field (in the second frame part of the image is already in focus). In the next two frames, the face is in focus. In the middle row, the white areas (mask values equal to 1), which are associated with the eyes or hair (fringe, moustache, or goatee), are visible. In the areas where there are no borders, the mask does not return positive values (1). The bottom row shows how the left eye regions are better defined in these two frames. When the person’s face leaves the depth of field, the whole face is out of focus again.

In tests with the system, it always detects eyes that are correctly focused, discarding a large number of unfocused eyes. Several image sequences are recorded and used for validation. Of these sequences, 97% of the eye detections are correctly discarded because they are out of focus. The proposed design aims to ensure that no eye that may be in focus is discarded, so the selection of the threshold can be considered conservative. This is the reason why, occasionally, images of out-of-focus eyes are allowed to pass to the iris pattern segmentation and normalisation phase. These images are discarded by the iris recognition module.

## 5. Conclusions and Future Work

This paper details how to design a defocus blur estimation core on the PL part of an MPSoC to allow an eye detection system to discard in real-time those eye images that are out of focus. The design is implemented on an AMD/Xilinx Zynq${}^{\mathrm{TM}}$ UltraScale+${}^{\mathrm{TM}}$ XCZU4EV platform by converting the C/C++ code to hardware logic core through Vitis HLS. The core design is optimised by using different optimisation directives to reduce latency. Thus, the proposed real-time eye detection system can correctly discard out-of-focus detected eye images but also process 57 images with 4096 × 4096 size per second. Significantly, when integrated in an IAAD recognition system, the defocus blur estimation core allows the system to discard 97% of the detected eye images.

Future work focuses on (1) implementing the next steps of the iris recognition system (iris pattern segmentation and normalisation) in the MPSoC, using the resources that are not yet being used (in the PL, but also in the PS, such as the GPU or the dual-core Cortex-R5); (2) further exploiting the use of the optimisation directives provided by Vitis HLS to improve the current proposed framework; and (3) adding the framework with the cores to implement an iris presentation attack detection system (iPAD).

## Figures and Tables

**Table 1:** Total resource utilisation for the DEFOCUS core (Daugman’s kernel). FF stands for flip flops, LUT for look-up tables, and DSP48E for digital signal processing elements. The DSP48E combines an 18-bit by 25-bit signed multiplier with a 48-bit adder and programmable mux to select the adder input.

Name	BRAM_18K	DSP48E	FF	LUT	URAM
DSP	–	–	–	–	–
Expression	–	–	0	2	–
FIFO	0	–	65	332	–
Instance	2	1	2606	4193	1
Memory	–	–	–	–	–
Multiplexer	–	–	–	–	–
Register	–	–	–	–	–
Total	2	1	2671	4527	1
Available	256	728	175,680	87,840	48
Utilisation (%)	0	0	1	5	2

**Table 2:** Total resource usage for the whole system.

Name	BRAM_18K	DSP48E	FF	LUT	URAM
Classifier	81	582	34,645	25,740	0
Defocus	2	1	2671	4527	1
Total	83	583	37,316	30,267	1
Available	256	728	175,680	87,840	48
Usage (%)	32	80	21	34	2

**Table 3:** Results obtained when evaluating convolution kernels using the Clarkson LivDet2013 [34].

Convolution Kernel	Groups 1–5		Groups 2–4		Group 3		Threshold Value
	Mean	Std	Mean	Std	Mean	Std	
Daugman [25]	2284.74	952.20	7044.57	5794.04	27,411.10	12,113.73	14,067.99
Wei et al. [11]	108.70	75.61	244.81	211.72	1206.34	599.93	531.47
Kang and Park [29]	194.31	131.00	450.28	399.91	2222.65	1132.99	969.92
Wan et al. [30]	51.39	16.67	67.79	29.95	186.91	83.30	100.68

## Data Availability

Not applicable.
